# In Vivo Allergen-Activated Eosinophils Promote Collagen I and Fibronectin Gene Expression in Airway Smooth Muscle Cells via TGF-*β*1 Signaling Pathway in Asthma

**DOI:** 10.3390/ijms21051837

**Published:** 2020-03-06

**Authors:** Ieva Janulaityte, Andrius Januskevicius, Virginija Kalinauskaite-Zukauske, Ieva Bajoriuniene, Kestutis Malakauskas

**Affiliations:** 1 Laboratory of Pulmonology, Department of Pulmonology, Lithuanian University of Health Sciences, LT-44307 Kaunas, Lithuania; andrius.januskevicius@lsmuni.lt (A.J.); kestutis.malakauskas@lsmuni.lt (K.M.); 2 Department of Pulmonology, Lithuanian University of Health Sciences, LT-44307 Kaunas, Lithuania; virginija.kalinauskaite@lsmuni.lt; 3 Department of Immunology and Allergology, Lithuanian University of Health Sciences, LT-44307 Kaunas, Lithuania; ieva.bajoriuniene@lsmuni.lt

**Keywords:** eosinophil, TGF-*β*1 signaling, airway smooth muscle cells, extracellular matrix proteins, bronchial allergen challenge, collagen I, fibronectin

## Abstract

Eosinophils infiltration and releasing TGF-*β*1 in the airways has been implicated in the pathogenesis of asthma, especially during acute episodes provoked by an allergen. TGF-*β*1 is a major mediator involved in pro-inflammatory responses and fibrotic tissue remodeling in asthma. We aimed to evaluate the effect of in vivo allergen-activated eosinophils on the expression of *COL1A1* and *FN* in ASM cells in asthma. A total of 12 allergic asthma patients and 11 healthy subjects were examined. All study subjects underwent bronchial challenge with *D. pteronyssinus* allergen. Eosinophils from peripheral blood were isolated before and 24 h after the bronchial allergen challenge using high-density centrifugation and magnetic separation. Individual co-cultures of blood eosinophils and immortalized human ASM cells were prepared. The TGF-*β*1 concentration in culture supernatants was analyzed using ELISA. Gene expression was analyzed using qRT-PCR. Eosinophils integrins were suppressed with linear RGDS peptide before co-culture with ASM cells. Results: The expression of *TGF-β1* in asthmatic eosinophils significantly increased over non-activated asthmatic eosinophils after allergen challenge, *p* < 0.001. The TGF-*β*1 concentration in culture supernatants was significantly higher in samples with allergen-activated asthmatic eosinophils compared to baseline, *p* < 0.05. The effect of allergen-activated asthmatic eosinophils on the expression of *TGF-β1*, *COL1A1*, and *FN* in ASM cells was more significant compared to non-activated eosinophils, *p* < 0.05, however, no difference was found on *WNT-5A* expression. The incubation of allergen-activated asthmatic eosinophils with RGDS peptide was more effective compared to non-activated eosinophils as the gene expression in ASM cells was downregulated equally to the same level as healthy eosinophils.

## 1. Introduction

Allergic asthma (AA) is a chronic inflammatory condition of the airways characterized by a type 2 inflammation with prominent eosinophilic infiltration in the bronchial mucosa [1,2]. When aeroallergen gets to the sensitized airway, the immune response quickly takes action. Firstly, the inflammation during the early phase starts with bronchoconstriction that clinically manifests as airway hyperreactivity [3]. Later, the increased production of mucus, vasodilatation, as well as vascular permeability occurs. Repeatedly inhaled allergens through various mediators stimulate eosinophils migration to airways that cause inflammation and edema [4].

Eosinophils recruitment from the bloodstream to inflamed tissues depends on circulating eosinophils becoming activated, which leads to eosinophil arrest on activated endothelium, extravasation, and continued movement through the bronchial tissue by interaction with extracellular matrix (ECM) [5]. Airway eosinophilia is one of the main features of asthma pathogenesis that leads to the changed microenvironment, causing airway remodeling [6]. Airway remodeling refers to the structural changes and activation of airway smooth muscle (ASM) cells and fibroblasts. Structural changes include excessive repair processes followed by repeated airway injury, including the increased deposition of several ECM proteins such as collagens and fibronectin in the reticular basement membrane and bronchial mucosa as well as increased ASM mass, goblet-cell hyperplasia, and neoangiogenesis [7]. The main producers of ECM proteins are pulmonary structural cells such as ASM cells and fibroblasts. ECM proteins contribute to the tissue structure and elasticity, which are seen unbalanced in asthma [8,9]. ECM can affect the behavior of the structural cell in lung tissue. The role of cell–matrix interactions represents an area for active investigation on the ability of the lung matrix to prime the structural pulmonary cells. Additionally, ECM proteins are responsible for ASM cell migration, contractility, proliferation in asthma [10,11].

Chronic inflammation is caused by activated inflammatory and structural cells that secrete various mediators. TGF-*β* plays a central role in the complex relationship between the activation of the inflammatory cascade in the airways and suppression of T cell immune function [12,13]. TGF-*β*1 is secreted by fibroblasts, endothelial cells, airway epithelial cells, vascular, and ASM cells. However, migrated inflammatory cells, such as eosinophils, are the rich source of fibrogenic factors, particularly TGF-*β*1 [1,5]. TGF-β1 is involved in increased expression of ECM proteins, tissue fibrosis, mucus production, as well as promotes the proliferation of ASM cells and fibroblasts [14,15,16]. It was shown that TGF-*β* can act differently depending on the situation—it can be an anti- or pro-inflammatory cytokine [17]. As an anti-inflammatory cytokine TGF-*β* is capable of regulating the proliferation and activation of B and T lymphocytes, deactivating macrophages [18,19,20]. At the same time, TGF-*β* is known for pro-inflammatory properties as it can participate in chemotaxis of eosinophils, T lymphocytes, B lymphocytes, neutrophils, induce proliferation of fibroblasts, suppress apoptosis of eosinophils, T lymphocytes, and neutrophils in asthma [17]. Additioally, TGF-*β* affects airway structural cells, such as epithelial cells, ASM cells, and pulmonary fibroblasts [21]. Once structural cells are activated the chain reaction of responses that lead to airway remodeling, including increased ECM production starts via the activated TGF-*β*–Smad signaling pathway. However, little is still known regarding the effect of eosinophils on the ASM cell production of main ECM proteins such as collagen I and fibronectin. In our previous study, it was shown that WNT-5A ligands may be the key regulators of increased ASM cell proliferation and gene expression of ECM proteins in ASM cells [22].

We hypothesized that allergen-activated eosinophils might more intensively affect gene expression of ECM proteins as *COL1A1* and *FN* in ASM cells via activated TGF-*β*1 signaling. For this purpose, we used the bronchial allergen challenge for eosinophil activation in vivo.

## 2. Results

### 2.1. Characteristics of the Study Population

Twenty-three non-smoking adults (nine men and 14 women) were included in the study: 12 patients with AA and 11 healthy subjects (HS). All study participants were non-smokers and with normal lung function at baseline. No significant age and sex differences were documented when both groups were compared. Atopy was demonstrated only in subjects with AA. At the baseline, AA patients had a significantly higher peripheral blood eosinophil count compared with the HS. Twenty-four hours after bronchial allergen challenge blood eosinophil significant increase was only in AA patients, but not in HS (Table 1).

Only nine AA patients and seven HS sputum samples were evaluated, as samples with more than 20% of epithelial cells were excluded. Cell viability and eosinophil count were significantly higher at the baseline in the AA group compared to the HS, *p* < 0.05. Allergen challenge significantly increased sputum cells’ viability as well as eosinophil count in AA compared to baseline, *p* < 0.05 (Table 1).

### 2.2. TGF-β1 Expression in Eosinophils and Airway Smooth Muscle Cells

**Figure 1:**
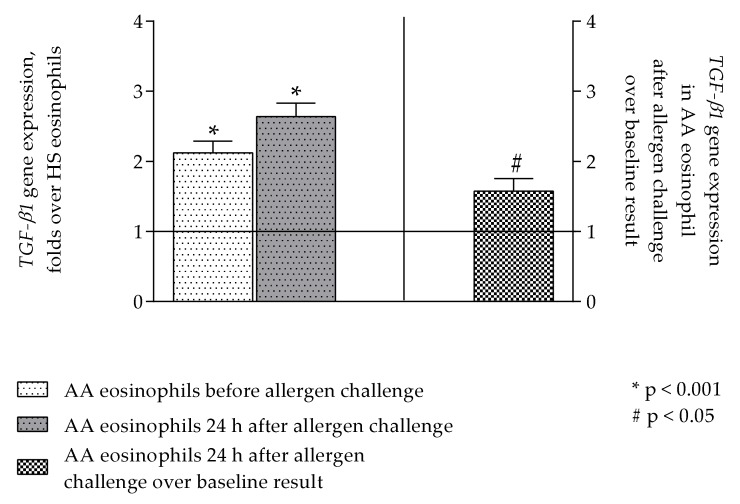
*TGF-β1* expression in asthmatic eosinophils before and 24 h after allergen challenge. Data represented as mean ± SEM evaluated as folds over healthy subjects’ eosinophils and as folds 24 h after allergen challenge over baseline asthmatic eosinophils. *TGF-β1*–transforming growth factor β1 gene; *n* = 12, * *p* < 0.001 comparing with HS group; # *p* < 0.05 comparing with eosinophils before allergen challenge, *n* = 12. Statistical analysis—Wilcoxon matched-pairs signed-rank test for analysis between the dependent groups; Wilcoxon signed-rank test for analysis against control ASM cells.

*TGF-β1* expression was evaluated in blood eosinophils before and 24 h after bronchial allergen challenge. *TGF-β1* expression in asthmatic eosinophils was evaluated by folds over healthy eosinophils. Expression of *TGF-β1* was significantly increased in AA patients’ eosinophils compared to healthy eosinophils, *p* < 0.001. Twenty-four hours after allergen challenge with *Dermatophagoides pteronyssinus* (*D. pteronyssinus*) tendency remained as asthmatic eosinophils had significantly higher *TGF-β1* expression compared to HS, *p* < 0.001. The gene expression was significantly higher 24 h after bronchial allergen challenge for 1.58 ± 0.18 folds over non-activated asthmatic eosinophils, *p* < 0.05 (Figure 1) while allergen challenge with allergen did not affect *TGF-β1* expression in healthy eosinophils (data not shown).

**Figure 2:**
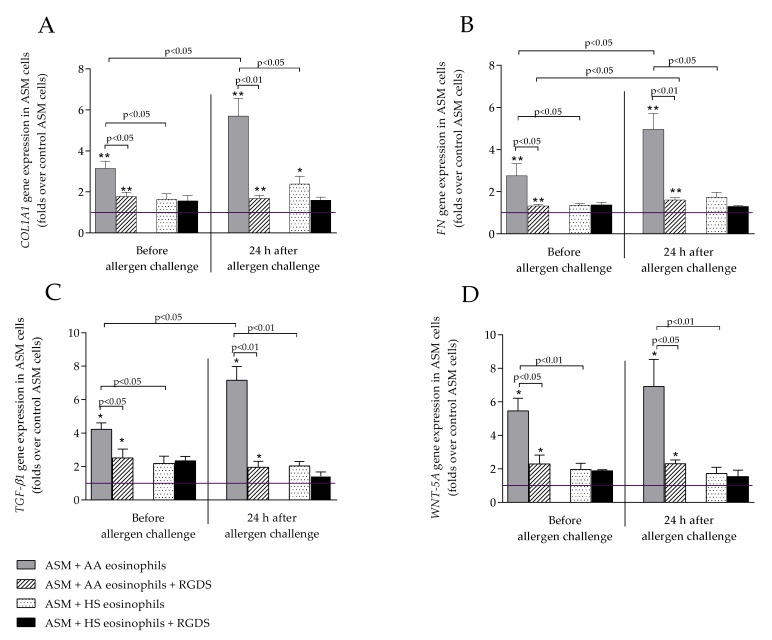
*COL1A1* (**A**), *FN* (**B**), *TGF-β1* (**C**), and *WNT-5A* (**D**) expression in ASM cells after combined culture with eosinophils before and 24 h after allergen challenge. Data represented as mean ± SEM evaluated as folds over control ASM cells that were not incubated with eosinophils. AA—allergic asthma; ASM—airway smooth muscle cells; *COL1A1*—collagen I A 1 gene; *FN*—fibronectin gene; HS—healthy subject; RGDS—arginyl-glycyl-aspartyl-serine peptide (Arg-Gly-Asp-Ser); *TGF-β1*—transforming growth factor *β*1 gene; *WNT-5A*—wingless/integrase-1-5A gene. * *p* < 0.05 compared to control ASM cells; ** *p* < 0.01 compared to control ASM cells; AA *n* = 12, HS *n* = 11. Statistical analysis—Mann–Whitney U test for analysis between AA and HS; Wilcoxon matched-pairs signed-rank test for analysis between the dependent groups; Wilcoxon signed-rank test for analysis against control ASM cells. Lines connect comparison groups with *p*-value denoting the significant difference in pair-wise comparisons.

Expression of *TGF-β1* was significantly increased in ASM cells after incubation with AA eosinophils compared to healthy eosinophils at the baseline, accordingly 4.23 ± 0.38 vs. 2.18 ± 0.44 folds over control ASM cells, *p* < 0.05, and after in vivo eosinophil activation with specific allergen the effect of asthmatic eosinophils to *TGF-β1* expression in ASM cells was even stronger compared to effect of healthy eosinophils, accordingly 7.16 ± 0.82 vs. 2.04 ± 0.26 folds over control ASM cells, *p* < 0.01 (Figure 2C). The allergen challenge significantly increased asthmatic eosinophil effect to *TGF-β1* expression in ASM cells, accordingly 7.16 ± 0.82 vs. 4.23 ± 0.38 folds over control ASM cells, *p* < 0.05, however, had no significant effect to HS eosinophils.

### 2.3. TGF-β1 Concentration in Culture Supernatants

**Figure 3:**
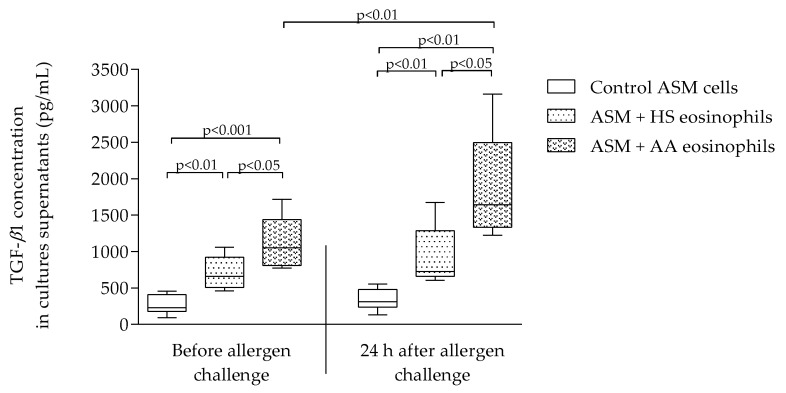
TGF-*β*1 concentration in supernatants of control ASM cell culture, ASM cells with HS eosinophils as well as ASM cells with AA eosinophils combined cultures before and 24 h after allergen challenge. Data represented as median (range). AA—allergic asthma; ASM—airway smooth muscle cells; HS—healthy subject; TGF-*β*1—transforming growth factor *β*1; control ASM cells that were not incubated with eosinophils, AA *n* = 8, HS *n* = 7. Statistical analysis—Mann–Whitney U test for analysis between control ASM cells, AA and HS; Wilcoxon matched-pairs signed-rank test for analysis between the dependent groups. Lines connect comparison groups with *p*-value denoting the significant difference in pair-wise comparisons.

TGF-*β*1 concentration was significantly increased in supernatants after incubation with asthmatic eosinophils before allergen challenge compared to healthy eosinophils, accordingly 1052 (772–1717) pg/mL vs. 662 (459–1061) pg/mL, *p* < 0.05. The same tendency was found 24 h after challenge: allergen-activated eosinophils significantly increased the concentration of TGF-*β*1 in culture supernatants compared to healthy eosinophils, accordingly 1643 (1224–3160) pg/mL vs. 725 (606–1673) pg/mL, *p* < 0.05. The in vivo allergen-activated asthmatic eosinophils significantly increased TGF-*β*1 concentration in culture supernatants compared to non-activated asthmatic eosinophils, accordingly 1643 (1224–3160) pg/mL vs. 1052 (772–1717) pg/mL, *p* < 0.05. Data presented in Figure 3.

### 2.4. WNT-5A Expression in Airway Smooth Muscle Cells

*WNT-5A* expression was significantly increased in ASM cells after incubation with asthmatic eosinophils compared to healthy eosinophils at the baseline, accordingly 5.64 ± 0.99 vs. 1.76 ± 0.44 folds over control ASM cells, *p* < 0.01 (Figure 2D). The same tendency was 24 h after allergen challenge: allergen-activated asthmatic eosinophils significantly increased *WNT-5A* expression in ASM cells compared to HS eosinophils, accordingly 6.91 ± 0.25 vs. 2.26 ± 0.69 folds over control ASM cells, *p* < 0.01. However, there was no statistically significant difference between allergen-activated and non-activated asthmatic eosinophil effect to *WNT-5A* expression in ASM cells.

### 2.5. COL1A1 and FN Expression in Airway Smooth Muscle Cells

Before allergen challenge asthmatic eosinophils significantly increased *COL1A1* and *FN* expression in ASM cells compared to healthy eosinophils, accordingly 3.15 ± 0.36 vs. 1.64 ± 0.27 folds over control ASM cells, *p* < 0.05, and 2.75 ± 0.59 vs. 1.35 ± 0.07 folds over control ASM cells, *p* < 0.05. 24 h after allergen challenge the similar tendency remained: allergen-activated asthmatic eosinophils significantly increased *COL1A1* and *FN* expression compared to healthy eosinophils, accordingly 5.70 ± 0.86 vs. 2.39 ± 0.37 folds over control ASM cells, *p* < 0.05; and 4.96 ± 0.76 vs. 1.73 ± 0.23 folds over control ASM cells, *p* < 0.01; and compared to baseline result (for *COL1A1* expression −5.70 ± 0.86 vs. 3.15 ± 0.36 folds over control ASM cells, *p* < 0.05, and for *FN* expression −4.96 ± 0.76 vs. 2.75 ± 0.59 folds over control ASM cells, *p* < 0.05). Data presented in Figure 2A,B.

### 2.6. Suppression of Eosinophil Integrins with RGDS Peptide

Non-specific suppression of integrins on asthmatic eosinophil surface by incubating them with arginyl-glycyl-aspartyl-serine (Arg-Gly-Asp-Ser) peptide (RGDS) significantly downregulated gene expression of all four genes to healthy eosinophil gene expression level in ASM cells. Integrins’ suppression only affected asthmatic eosinophils—before and 24 h after bronchial allergen challenge on all four—*TGF-β1*, *WNT-5A*, *COL1A1*, and *FN*-expression in ASM cells after incubation with eosinophils. Gene expression was significantly downregulated before (accordingly *COL1A1* 3.15 ± 0.36 vs. 1.78 ± 0.21 folds over control ASM cells, *p* < 0.05; *FN* 2.75 ± 0.59 vs. 1.32 ± 0.08 folds over control ASM cells, *p* < 0.05; *TGF-β1* 4.23 ± 0.38 vs. 2.51 ± 0.53 folds over control ASM cells, *p* < 0.05; *WNT-5A* 5.64 ± 0.99 vs. 2.87 ± 0.51 folds over control ASM cells, *p* < 0.05) and 24 h after allergen challenge (accordingly *COL1A1* 5.70 ± 0.86 vs. 1.69 ± 0.14 folds over control ASM cells, *p* < 0.01; *FN* 4.96 ± 0.76 vs. 1.61 ± 0.10 folds over control ASM cells, *p* < 0.01; *TGF-β1* 7.16 ± 0.82 vs. 1.86 folds over control ASM cells, *p* < 0.01; *WNT-5A* 6.91 ± 1.86 vs. 2.32 ± 0.25 folds over control ASM cells, *p* < 0.05). However, the expression of selected genes after the suppression of integrins on the eosinophil outer membrane remained significantly higher compared to control ASM cells that were not incubated with eosinophil and did not differ from the effect of healthy eosinophil (Figure 2A–D).

## 3. Discussion

In this study, we showed that eosinophil activation with *D. pteronyssinus* allergen in vivo increased TGF-β1 gene expression in asthmatic eosinophils and enhanced their effect on *TGF-β1*, *COL1A1*, and *FN* expression in ASM cells. However, asthmatic eosinophils increased *WNT-5A* expression in ASM cells equally before and 24 h after bronchial allergen challenge. The addition of RGDS peptide reduced asthmatic eosinophils effect on gene expression in ASM cells to a healthy eosinophils effect level.

AA is associated with eosinophilic airways inflammation [23]. The maturation of eosinophils is activated in bone marrow, and they are recruited to the airways to cope with environmental triggers that lead to inflammation. These processes are associated with type 2 inflammation as the airway epithelial cells release alarmins such as interleukin (IL) 25, IL-33, and thymic stromal lymphopoietin (TSLP), which activate innate and humoral immune system [24,25,26,27]. Inhaled allergens sensitize dendritic cells that stimulate the proliferation of T helper type 2 (Th2) cells and subsequent release of cytokines that include IL-4, IL-5, IL-13 [4,27]. IL-5 is crucial for eosinophils maturation in the bone marrow, while other cytokines are responsible for eosinophils release to blood flow. CC chemokine receptor 3 (CCR3) on the outer eosinophil membrane and eotaxins regulates the migration to inflamed airway tissues through permeable blood vessel walls [28,29,30]. Additionally, in the migration processes the eosinophil outer membrane receptors and integrins play a critical role. It was shown that eosinophils have various integrins, but the two heterodimers (*α*M*β*2 and *α*4*β*1) are the most important integrins in migration as they recognize the adhesion molecules of pulmonary structural cells and are dysregulated in asthma [31]. In our previous publications, we showed that AA eosinophils had higher outer membrane integrins *α*M*β*2 and *α*4*β*1 gene expression compared to HS [27,32]. It shows that AA eosinophils are more activated as the molecules that are responsible for adherence are upregulated, and it helps eosinophils to migrate through the wall of vessels and continue the movement to bronchial tissue interacting with pulmonary structural cells and ECM proteins. Previously we showed that in vivo allergen-activated eosinophils demonstrate a higher adhesion, viability, and pro-proliferative effect on ASM cells and pulmonary fibroblasts compared to non-activated eosinophils [33]. A possible limitation of this study is that we used peripheral blood eosinophils instead of airway tissue eosinophils. However, peripheral blood eosinophils taken before extravasation to lung tissues are already in an active state. 

Remodeling and repair processes in the lung are associated with permanent structural and functional changes in homeostatic cellular and physiological state. These processes include dysregulation of expression and increased deposition of ECM proteins, cellular differentiation to more active cell subtypes, disbalance of apoptosis and necrosis, increased cell proliferation [34]. Collagen I and fibronectin together are the main ECM proteins that are responsible for cell behavior such as proliferation, increased survival under stress, and migration [35,36,37,38,39]. Collagens are known to modulate cell behavior and function directly or via interactions with integrins and growth factor-mediated mitogenic pathways [40]. *COL1A1* codes collagen I α chain molecule. Collagen I forms fibrils that are responsible for scarring and tissue repair. In asthma studies, collagen I was shown to be accumulated more in asthmatic airways compared to healthy airways and is associated with increased mass of ASM bundles [41]. Another ECM protein that changes cell behavior is fibronectin. It was shown that fibronectin expression in asthmatic airways was higher than in healthy airways [42]. However, it remains unclear if the ASM cells themselves are different, or the inflammatory cells, such as eosinophils, act differently depending on the severity and type of asthma. In the study, we showed that *COL1A1* and *FN* expression in healthy ASM cells was increased after incubation with asthmatic allergen-activated eosinophils compared to baseline (Figure 2A,B). It shows that eosinophils are capable of changing ASM cell activity and increase gene expression of ECM proteins. Additionally, the addition of RGDS peptide on activated eosinophil significantly downregulated the gene expression of these ECM proteins (Figure 2A,B). However, the similar changes in the airways, only in vivo, were shown in a study where several ECM proteins such as collagen I, fibronectin, elastin as well as matrix metalloproteinases (MMP) 9 and 12 were increased in large and small airways during an autopsy in fatal asthma patients [43]. The possible limitation of our study is that we evaluated the changes in gene expression but not in the protein level. It is stated that the quantity of transcript may not always correlate with the protein level. However, Antonis Koussounadis et al. in 2015 showed that differentially expressed mRNA correlates significantly better with their protein product than non-differentially expressed mRNA [44]. It means that under different conditions, for example comparing disease affected patients with healthy subejcts, the changes in mRNA correlate with the protein level. In our study, the ASM cells incubated with AA and HS eosinophils have significant differences in gene expression. Studies with biopsies from asthmatic subjects showed that the gene expression as well as protein expression of TGF-*β,* collagen I and fibronectin were significantly increased compared to HS [45,46,47]. Therefore, we suggest that our study data, as well as this in vitro model, might be helpful in understanding the asthma pathogenesis in vivo.

Integrins are α and *β* subunits containing cellular receptors found on cells’ outer membranes. Cell–ECM and cell–cell interaction is generally controlled by integrins, and this interaction is required not only for eosinophils rolling and tethering but also for their activation [48]. We previously demonstrated that adhesion of eosinophils to pulmonary structural cells or ECM proteins increases their viability [33]. Eosinophils express seven integrins heterodimers, and each type interacts with its own set of ligands, which may be deposited in ECM or as a counter-receptor on other cells [31]. Eosinophils do not express RGD-binding integrins; however, several studies showed that in the fully activated state of eosinophils, the *α*4*β*1 and *α*M*β*2 integrins can recognize RGD motifs [48,49,50,51]. It is probably because integrins that do not bind via an RGD-motif on their ligands seem to have RGD-binding structures within their ligand-binding pockets [52]. The linear form of RGD containing peptides demonstrates very little selectivity among the integrin receptors [32]. We used RGDS peptide to suppress the integrins and in that way reduce the intensity of eosinophil adhesion. [53]. Our study showed that suppression of eosinophil integrins downregulated target gene expression; however, it remained significantly higher than in control ASM cells. We presume that it may be caused by increased *TGF-β1* expression. In the previous study we showed that suppression of eosinophil integrins with RGDS peptide reduced TGF-*β* concentration in combined cultures between ASM cells and eosinophils [32]. However, TGF-*β* concentration remained significantly higher compared to the healthy eosinophils effect. The way that the suppression of eosinophil integrins contributes to the TGF-*β*1 and ECM protein expression can be explained. Firstly, suppression may affect eosinophils viability due to decreased adhesive intensity. As the eosinophil lifetime is prolonged, it can produce various mediators that participate in the promotion of gene expression, but the suppression of eosinophil adherence reduces their viability thus reducing the possible impact for airway remodeling. Furthermore, suppressed eosinophils may produce less mediators, proteinases, and reactive oxygen species (ROS) leading to decreased activation of TGF-*β*1. However, this part needs to be clarified more in future work. A possible limitation of the study is that residual RGDS that may be left after the washing step could be added together with eosinophils into the culture well. The residual RGDS could bond to the ASM cell integrins or expressed fibronectin, thus reducing the number of eosinophil attachment sites and can bind to RGDS binding integrins on the ASM cell outer membrane thus changing cell behavior. However, the residual amount of RGDS could not dramatically change results as the eosinophils were washed several times. 

TGF-*β* is a multifunctional cytokine that, depending on the disease, participates in stimulation or inhibition of cell proliferation, controls ECM synthesis and degradation as well as cell and tissue response to injury [54]. This cytokine is one of the key players in airway remodeling in asthma [55]. TGF-*β* is synthesized by inflammatory and lung structural cells—such as eosinophils, fibroblasts, and ASM cells. TGF-*β* is a major mediator in asthma, and a number of secondary anti-inflammatory effects result from the autocrine/paracrine actions of the TGF-*β* production. It was shown that TGF-*β* transcription is regulated by p38, extracellular signal-regulated kinases (ERK), mitogen-activated protein kinases (MAPK), and c-Jun N-terminal kinases (JNK) signaling pathways [56]. Eotaxins induce eosinophil degranulation and release of biologically active mediators through the activation of ERK2 and p38 MAPK signaling [57]. In the previous study, we showed that the level of eotaxins was increased in serum collected from AA patients compared to HS [27]. We presume that an increased level of eotaxins induces TGF-*β* transcription in blood eosinophils. Furthermore, it was shown that the p38 and ERK signaling pathways are promoting TGF-*β* transcription as they are activated in asthmatic ASM cells [58]. 

TGF-*β* is secreted as the latent complex that accumulates in ECM and requires activation to be a functionally active molecule. The proteases such as MMP-2 and MMP-9, ROS, pH, and integrins contribute to the liberation of active TGF-*β* from ECM. Eosinophils produce increased levels of MMP-9, ROS as well as TGF-*β* in asthma [59,60,61]. Prolonged viability of eosinophils after migration to airways and adhesion to ECM and/or ASM cells creates conditions for the secretion of mediators by eosinophils [33]. It was shown that ASM cells can activate TGF-*β*1 via *α*V*β*5 integrins—specifically through the *β*5 cytoplasmic domain [62]. The expression of *α*V*β*5 integrin heterodimer in ASM cells is increased in asthma, and the blocking of this integrin prevents TGF-*β* activation. The TGF-*β* signaling pathway is a complex mechanism of the phosphorylation of downstream Smad proteins, comprising the receptor-regulated Smad 2/3, and the co-mediator Smad 4 and the inhibitory Smad 7. Activated Smad complexes translocate to the nucleus to upregulate the transcription of ECM proteins genes such as *COL1A1* and *FN* through a Smad-dependent mechanism [54,63,64,65]. In the murine study model, it was shown that the antibody of TGF-*β*1 prevents phosphorylation of Smad2 in prolonged allergen challenge-induced asthma [66]. Additionally, the anti-TGF-*β* antibody resulted in the phosphorylated Smad2 signaling inhibition, reduced mucus production, and ECM deposition in the airway wall, as well as decreased ASM cell proliferation in mice [66]. These findings suggest that TGF-*β* may be responsible for the increased expression of phosphorylated Smad2 in the airways of asthmatics in humans also.

Previously, it was found that the TGF-β1 levels in serum of atopic asthma patients are increased compared to non-atopic control subjects [67]. In the biopsies taken from asthmatic bronchi, it was found that about 70–80% of all TGF-*β* expressing cells are eosinophils, showing the link between TGF-*β* expression and airway inflammation [45,55]. In our study, we showed that allergen challenge activates asthmatic eosinophils as the expression of TGF-*β*1 is significantly higher 24 h after allergen challenge compared to baseline (Figure 1). The TGF-*β*1 protein concentration in culture supernatants was significantly higher in those samples that were obtained from combined cultures between ASM cells and asthmatic eosinophils compared to the effect of healthy eosinophils (Figure 3). Allergen-activated eosinophils significantly increased TGF-*β*1 concentration compared to baseline. The TGF-β1 concentration in supernatants matched gene expression results. Previously, it was found that in murine, the Th17 cells also play an important role at the asthma pathogenesis by producing IL-17, IL-23, IL-25, which results in airway inflammation [68,69]. Studies revealed that the Th17 cells number in human peripheral blood and mice lung tissue were increased after allergen challenge with *D. pteronyssinus* [70,71]. The following study showed that eosinophils stimulation by IL-17A and IL-17F promotes these cells to secrete TGF-*β* [72]. Allergen-activated eosinophils damage the airways, and it leads to remodeling through various signaling pathways. One of these signaling pathways is TGF-*β*1/WNT-5A. Wingless/Integrase-1 (WNT) is a signaling pathway associated with normal various organ morphogenesis in embryogenesis and lung repair in adults. Some studies show the WNT signaling pathway has an important role in asthma pathogenesis [22,73]. Several growth factors, including TGF-β1, are responsible for non-canonical WNT-5A signaling pathway activation through *β*-catenin directly as well as by autocrine increased production of WNT ligands. Still, it is not known how WNT-5A signaling is changed during acute asthma, but there is an increased expression of WNT-5A in asthmatic ASM cells. These changes have been linked to type 2 inflammation [74]. Additionally, it was shown that non-canonical WNT-5A signaling is important in TGF*-β* induced ECM production by ASM cells in asthma [75]. In this study, we showed that asthmatic eosinophils significantly increase *WNT-5A* expression in ASM cells (Figure 2D). As activated and non-activated asthmatic eosinophil increased *WNT-5A* expression without significant differences in the asthma group, we presume that the WNT-5A signaling pathway was not the major signaling pathway responsible for changes in ASM cell activity.

TGF-*β*1 as multifunctional cytokine has an important role in immune and stem cell regulation and differentiation, so it is a highly researched cytokine in the auto-immune, infectious diseases, as well as in cancer fields. Additionally, TGF-*β*1 is one of the growth factors that play a significant role in airway remodeling via increased ASM contractility and activity [76]. Burgess et al. showed that using corticosteroids or long-acting *β*2-agonists did not suppress TGF-*β* mediated ECM production in ASM cells [77]. Moreover, the corticosteroid itself may increase connective tissue growth factor (CTGF), collagen I, and fibronectin production. Blood eosinophils from asthmatics have significantly higher expression of TGF-*β*1 [78]. In the current study, TGF-*β*1 expression in asthmatic eosinophils and in ASM cells that were incubated with allergen-activated eosinophils was significantly increased (Figure 2C). This study shows that TGF-*β*1 may be the critical player in airway remodeling. Additionally, we showed that allergen-activated eosinophil effects could be managed using a RGDS peptide that non-specifically suppresses eosinophil adhesion to ASM cells and has a direct effect on them. Addition of RGDS peptide downregulated expression of *TGF-β1* in ASM cells (Figure 2C). We presume that remaining increased gene expression in ASM cells is due to increased allergen-activated eosinophil expression of *TGF-β1*.

Allergen-induced responses are associated with increased airway eosinophilia, which has been measured in induced sputum samples from AA patients [79]. In our study, patients with AA had a significantly greater sputum eosinophil count as well as increased blood eosinophil count at the baseline compared to HS. Twenty-four hours after bronchial allergen challenge with *D. pteronyssinus* allergen eosinophil count in sputum was significantly higher compared to asthma patients at the baseline (Table 1). Additionally, one of the most important features of AA patients was increased sputum cell viability that was significantly higher compared to HS, and after bronchial allergen challenge, viability percentage was even higher. It means that in the airways, there is not only more migrated eosinophils, but their apoptosis is reduced. In the previous study, we showed that blood eosinophils apoptosis from AA patients and allergic rhinitis patients were significantly lower compared to HS [59]. Similar results were shown in another study where asthma was phenotyping using sputum cell analysis or looking at the associations between sputum eosinophils apoptosis level and asthma severity [80]. Recent studies reported that blood and sputum eosinophilia are important factors for the prediction of asthma exacerbations [81]. Based on our study data, we state that avoiding the allergen is a rational part of an allergic asthma treatment plan that can help to reduce airway remodeling processes.

## 4. Materials and Methods

The research protocol was approved on 15 of November in 2016 by the Kaunas Regional Biomedical Research Ethics Committee of the Lithuanian University of Health Sciences with permission no. BE-2-13. The research study was registered in the US National Institutes of Health trial registry ClinicalTrials.gov with identifier NCT03388359.

### 4.1. Study Subjects

The study group consisted of 12 AA patients and the controls—11 HS aged between 18 and 65 years. AA patients were recruited from the Department of Pulmonology, Hospital of the Lithuanian University of Health Sciences. All study participants gave written informed consent. In the recruitment stage, all subjects were screened: they underwent a clinical examination, spirometry, methacholine challenge test, skin prick test, complete blood count.

The applied inclusion criteria for the AA group were inhaled steroid-free AA, approved with disease-specific symptoms and medical history more than one year with the mild-to-moderate course of the disease, positive skin prick test (≥3 mm) in response to house dust mites *D. pteronyssinus*, airway hyper-responsiveness to methacholine.

The applied inclusion criteria for the HS group were no use of medications, negative skin prick test; negative bronchial methacholine challenge test; no other chronic respiratory disease.

Exclusion criteria for both groups were defined as clinically significant permanent allergy symptoms, asthma exacerbation, or active airway infection one month prior to the study; use of oral steroids less than one month prior to the study; smoking.

### 4.2. Study Design

At the screening visit, inclusion/exclusion criteria were checked, and study subjects signed informed consents. After that, spirometry, methacholine challenge, and skin prick tests were performed. During the baseline visit, blood samples were collected, and bronchial allergen challenge with *D. pteronyssinus* was performed. The second study visit was scheduled 24 h later, and blood samples were re-taken.

**Figure 4:**
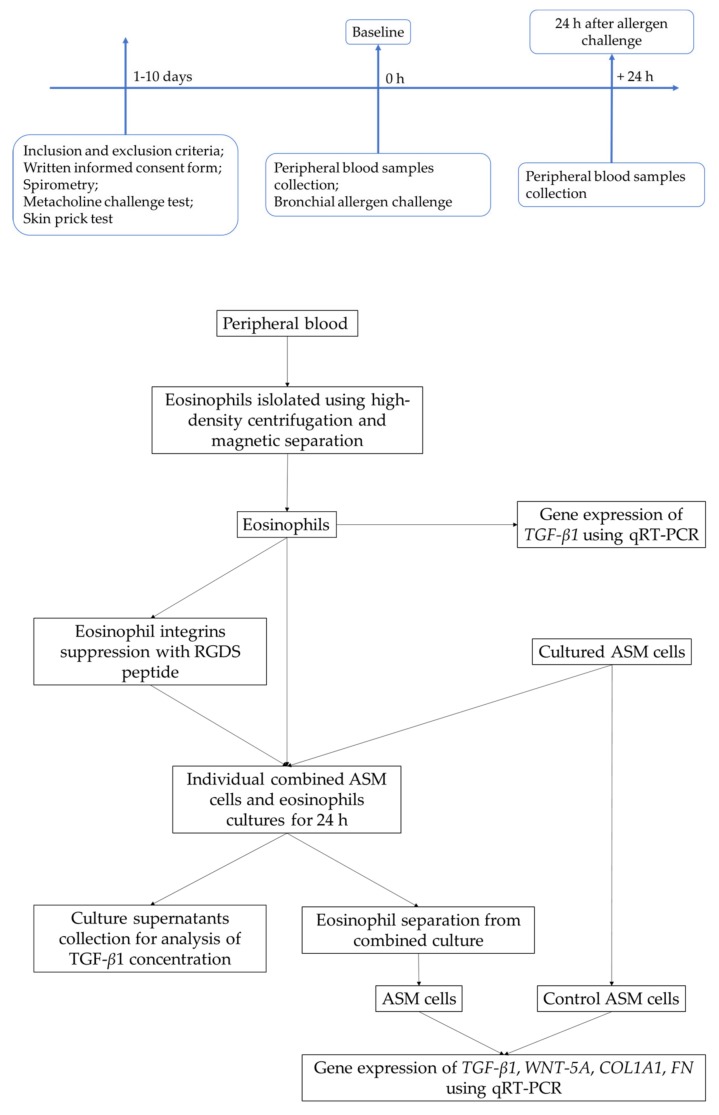
The flowchart of the study design: recruitment of study subjects, clinical examination, and experimental workflow. TGF-*β*1—transforming growth factor *β*1; *TGF-β1*—transforming growth factor *β*1 gene; *WNT-5A*—wingless integrase-1 5A gene; *COL1A1*—collagen I alpha 1 gene; *FN*—fibronectin; ASM—airway smooth muscle cells; RGDS—arginyl-glycyl-aspartyl-serine peptide (Arg-Gly-Asp-Ser); qRT-PCR—quantitative reverse transcription polymerase chain reaction.

A flow chart of the study design is presented in Figure 4.

### 4.3. Lung Function Testing

The lung function was evaluated according to baseline forced expiratory volume in 1 s (FEV_1_), forced vital capacity (FVC), and FEV_1_/FVC ratio using a Ganshorn spirometer (Ganshorn Medizin Electronic, Niederlauer, Germany). Baseline FEV_1_, FVC, as well as FEV_1_/FVC ratio, were recorded as the highest result of three reproducible measurements and compared with the predicted values matched for body height and weight, age, and sex according to the standardized methodology. Each of these values were repeatedly measured three times, and only the highest value FEV_1_ was taken for analysis.

### 4.4. Measurement of Airway Responsiveness to Methacholine

All study subjects underwent measurement of airway responsiveness to methacholine. The methacholine and allergen challenge tests were performed with a pressure dosimeter (ProvoX, Ganshorn Medizin Electronic, Germany). Aerosolized methacholine was inhaled with 2 min intervals starting with 0.0101 mg methacholine dose, increasing it step by step up to 0.121, 0.511, 1.31 mg of the total cumulative dose was achieved either until a 20% decrease in FEV_1_ from the baseline. The bronchoconstricting effect of methacholine after each dose was expressed as a percentage of decrease in FEV_1_ from the baseline values. The provocative dose of methacholine causing a ≥ 20% fall in FEV_1_ (PD_20M_) was calculated using the log dose-response curve by linear interpolation of the two adjacent data points.

### 4.5. Skin Prick Test

All study subjects were screened for allergies using standardized allergen extracts (Stallergenes S.A., Antony, France) by the skin prick test for the following allergens*: D. pteronyssinus*, *D. farinae*, cat and dog dandruff, five mixed grass pollen, mugwort allergen, birch pollen, *Alternaria*, *Aspergillus*, and *Cladosporium*. As a positive control, the histamine hydrochloride (10 mg/mL) was used, negative control—diluent (saline). The skin prick test was evaluated 15 min after application. The results of the test were considered as positive if the wheel diameter was ≥ 3 mm. Only patients sensitized to *D. pteronyssinus* were included in the study.

### 4.6. Bronchial Allergen Challenge

All study subjects underwent bronchial allergen challenge with *D. pteronyssinus* allergen (Stallergenes S.A., Antony, France). The allergenicity of allergen was evaluated by the index of reactivity (IR), which is not comparable to the other allergen units. First of all, the bronchoconstricting effect of nebulized saline was assessed, and after that aerosolized allergen was inhaled at 10-min intervals starting with 0.1 index of reactivity (IR) allergen concentration, increasing it step by step up to 1.0 IR/mL, 10.0 IR/m, 33.0 IR/mL, or a 20% decrease in FEV_1_ from the baseline was achieved.

### 4.7. Isolation of Eosinophils from Peripheral Blood

Peripheral blood was collected in dipotassium ethylenediaminetetraacetic acid (K2EDTA) vacutainers (BD Vacutainer^®^, Becton Dickinson U.K. ltd, Wokingham, UK) before and 24 h after bronchial allergen challenge. Samples were diluted 1:1 by adding 1× phosphate buffer saline (PBS). Then, the suspension was centrifuged using density gradient centrifugation as it was carefully layered over Ficoll-Paque (*ρ* = 1.077 g/mL) in tubes and centrifuged at 400 g for 30 min at 20 °C (Labmaster^®^ABC-CB200R, HANLAB Ltd., Cheongju, Korea). A top layer with mononuclear cells was removed. Granulocytes were separated using hypotonic lysis of erythrocytes. Later, the granulocytes pellet was resuspended in cold MACS buffer and incubated with Biotin-Antibody Cocktail as well as Micro-Beads for magnetic eosinophil separation from granulocytes using the manufacturer’s protocol (Miltenyi Biotec, Somerville, MA, USA). The manufacturer states and confirms that the eosinophils separation kit does not influence eosinophils viability, and separation efficiency is more than 97%. Eosinophils were counted using an automatic cell counter ADAM (Witec AG, Sursee, Switzerland)—eosinophils viability was found to be at least 98%.

The complete blood count test was performed on an automated hematology analyzer (Sysmex XE-5000, Sysmex Corporation, Kobe, Japan).

### 4.8. Airway Smooth Muscle Cell Culture

Healthy human ASM cells, immortalized by stable expression of human telomerase reverse transcriptase (hTERT), as described previously [82], were used for experiments. For all experiments, the same hTERT ASM cell line was used, thus avoiding changes in ASM activity and viability that could result from repeated thawing and passage. Cells were cultivated on plastic dishes with standard culture conditions of 5% CO_2_ in air at 37 °C with medium renewal every 48–72 h. For all experiments, cells were grown on plastic dishes in Dulbecco’s modified Eagle medium (DMEM) (GIBCO^®^; Life Technologies, Paisley, UK) supplemented with streptomycin/penicillin (2% *v*/*v*; GIBCO^®^; Life Technologies), amphotericin B (1% *v*/*v*; GIBCO^®^; Life Technologies), and fetal bovine serum (FBS) (GIBCO^®^; Life Technologies). Cells were serum-deprived in DMEM supplemented with antibiotics and insulin, transferrin, and selenium (ITS) reagent (GIBCO^®^; Life Technologies) before each experiment to stop ASM cell proliferation and avoid possible errors in gene expression analysis due to the effects of mediators of FBS in the growth medium.

### 4.9. Combined Culture of Airway Smooth Muscle Cells and Eosinophils

Isolated eosinophils were separated into two parts: one part was used as control eosinophils before and 24 h after allergen challenge; another part of eosinophils was used for experiments with eosinophil integrins suppression peptide arginine-glycine-aspartate-serine (Arg-Gly-Asp-Ser, RGDS by Sigma Aldrich, Merck KGaA, St. Louis, MS, USA) (Figure 4). Respectively, the amount of eosinophils suspended in the serum-free growth medium was taken, and the solution of RGDS was added to the final concentration of 0.125 mg/mL. Freshly isolated eosinophils with RGDS were incubated for 1 h at 37 °C. After incubation eosinophils were centrifuged, the serum-supplemented growth medium was removed and resuspended in a fresh serum-free growth medium.

For ASM cells, cultivation dishes with approximately 2 × 10^5^ cells were prepared, and combined cultures were made by adding suspension with 0.5 × 10^5^ isolated viable eosinophils to the ASM cells. To observe and visualize the cell growth, an inverted microscope (CETI Inverso TC100; Medline Scientific, Oxford, UK) was used.

The combined cultures of ASM cells and eosinophils were incubated for 24 h. After incubation, eosinophils were washed out using warm PBS ×1 (GIBCO, Life Technologies) incubating co-cultures for 5 min at 36.6 °C and gently tapping on dish sides. ASM cells were then collected and lysed for gene expression analysis.

### 4.10. RNA Isolation and Quantitive Real-Time PCR Analysis

For gene expression, the eosinophils were separated from ASM cells after 24 h of incubation. Total ribonucleic acid (RNA) was isolated according to the manufacturer’s instructions using the miRNeasy Mini Kit (Qiagen, Valencia, CA, USA). Quantitive real-time polymerase chain reaction (qRT-PCR) was performed in the 7500 Fast Real-Time PCR System using a PowerSYBR^®^Green RNA-to-CT™ 1-Step Kit (Applied Biosystems, Foster City, CA, USA) according to the manufacturer’s protocol.

Primers that were used to analyze gene expression are shown in Table 2.

### 4.11. The Concentration of TGF-β1 in Culture Supernatants Analysis

For TGF-*β*1 protein concentration in culture supernatants the Quantikine ELISA kit for Human TGF-*β*1 (R&D Systems^®^, Minneapolis, MN, USA) was used according to the instructions provided by manufacturers. The mean of the minimum detectable dose is 4.61 pg/mL. The TGF-*β*1 concentration was evaluated in control ASM cell culture supernatants; ASM cells and asthmatic eosinophils as well as ASM cells and healthy eosinophils cultures’ supernatants before and 24 h after bronchial allergen challenge. As the manufacturer notes, the Sample Activation Kit was used. The optical density was determined within the 30 min using a microplate reader set to 450 nm and for wavelength correction, the 540 nm wavelength was set to correct the optical imperfections in the plate. Every sample was done in two replicates. The results are shown as the median (range).

### 4.12. Sputum Induction, Processing, and Cell Analysis

The prepared sputum cytospins samples of induced sputum were prepared using a cytofuge instrument (Shandon Southern Instruments Inc, Sewickley, PA, USA) were stained by the May-Grünwald-Giemsa method for differential cell counts. Viability was calculated using ADAM-MC Automatic Cell Counter (NanoEnTek Inc, Mountain View, CA, USA). Cell differentiation was determined by counting approximately 500 cells in random fields of view under a light microscope, excluding squamous epithelial cells in four replicates. All samples with more than 20% of squamous epithelial cells were eliminated. The cells were identified by standard morphological criteria, nuclear morphology, and cytoplasmic granulation. Cell counts were expressed as percentages of total cells.

### 4.13. Statistical Analysis

Statistical analysis was performed by using GraphPad Prism 6 for Windows (Version 6.05, 2014; GraphPad Software, Inc., San Diego, CA, USA). The normality assumption of data was verified using the Shapiro–Wilks test. All the data were distributed not normally and were presented as the mean and standard error of the mean (SEM) or standard deviation (SD). The concentration of TGF-*β*1 in culture supernatants was presented as the median (range). Nonparametric tests were used because of a skewed distribution of the variables. The differences between two independents in data before and after bronchial challenge independent groups were evaluated for statistical significance by the Wilcoxon matched-pairs signed-rank test for analysis between dependent groups. Differences between two independent groups were evaluated using the Mann–Whitney U test for analysis between groups. Wilcoxon signed-rank test was used for gene expression analysis against the control ASM cells. Statistical significance was assumed when *p* < 0.05.

## 5. Conclusions

Increased activity and prolonged viability of eosinophils caused by allergens is one of the main causes of airway remodeling in AA. *TGF-β1* is the key regulator of airway structural cells’ function and production of ECM proteins, including collagen I and fibronectin. Increased *TGF-β1* expression in allergen-activated asthmatic eosinophils are responsible for increased *TGF-β1*, *COL1A1*, and *FN* expression in ASM cells. Suppression of allergen-activated eosinophil integrins downregulates *TGF-β1*, *COL1A1*, and *FN* expression in ASM cells to the healthy eosinophil effect level showing that eosinophils might affect and change ASM cell behavior directly via integrins as well as indirectly via eosinophil-derived TGF-*β*1.

## Figures and Tables

**Table 1:** Demographical and clinical data of study subjects.

	AA Patients, *n* = 12		HS, *n* = 11	
Age, median (range), years	28.5 (20–44)		26.0 (23–42)	
Sex, (male/female), *n*	4/8		5/6	
BMI, kg/m^2^	22.4 ± 2.6		24.0 ± 5.1	
Sensitization to *D. pteronyssinus*/*D. farinae*/birch/five grass mixture allergen, *n*	12/11/2/4		NR	
Wheel diameter by *D. pteronyssinus*, median (range), mm	5 (3–8)		NR	
PD_20M_, geometric mean (range), mg	0.09 (0.007–0.260)		NR	
PD_20A_, geometric mean (range), IR/mL	6.684 (1.631–9.403)		NR	
Maximum fall in FEV_1_after bronchial allergen challenge, mean % (min–max)	–31.2 (−52.1–−22.4)		−3.8 (−7.2–0.0)	
FEV_1_, % of predicted	99.0 ± 5.73		102.0 ± 7.05	
FEV_1_, L	3.69 ± 0.36		4.14 ± 0.54	
	Baseline	24 h after allergen challenge	Baseline	24 h after allergen challenge
Blood eosinophil count, ×10^9^/L	0.34 ± 0.11 * #	0.52 ± 0.30 #	0.15 ± 0.06	0.16 ± 0.04
Blood eosinophil count, %	7.08 ± 3.98 * #	8.63 ± 3.01 #	2.00 ± 1.05	2.51 ± 0.74
Sputum cell viability, % (AA *n* = 9, HS *n* = 7)	70.5 ± 5.34 * #	79.9 ± 11.2 #	51.5 ± 14.9	57.5 ± 12.3
Sputum eosinophil count, % (AA *n* = 9, HS *n* = 7)	5.5 ± 5.4 * #	13.3 ± 12.87 #	0.1 ± 0.2	0.5 ± 0.4

Data presented as a median (range), geometric mean (range), or mean ± SD. AA—allergic asthma; HS—healthy subjects; BMI—body mass index; PD_20M_—a provocative dose of methacholine causing a 20% drop in FEV_1_; IR—index of reactivity; PD_20A_—a provocative dose of allergen causing a 20% drop in FEV_1_; FEV_1_—forced expiratory volume in one second; NR—not responding.* −*p* < 0.05 compared to the result 24 h after allergen challenge; # −*p* < 0.05 compared to the HS group at the same visit.

**Table 2:** Sequences of primers used for gene expression analysis.

Gene	Forward 5′-3′	Reverse 5′-3′
*18S*	CGCCGCTAGAGGTGAAATTC	TTGGCAAATGCTTTCGCTC
*WNT-5a*	GGGTGGGAACCAAGAAAAAT	TGGAACCTACCCATCCCATA
*TGF-β1*	GTACCTGAACCCGTGTTGCT	GAACCCGTTGATGTCCACTT
*COL1A1*	TCGAGGAGGAAATTCCAATG	ACACACGTGCACCTCATCAT
*FN*	AGCCAGCAGATCGAGAACAT	TCTTGTCCTTGGGGTTCTTG

## Abbreviations

AA	Allergic asthma
ASM	Airway smooth muscle
BMI	Body mass index
CCR3	CC chemokine receptor 3
*COL1A1*	Collagen I alpha 1 gene
CTGF	Connective tissue growth factor
DMEM	Dulbecco’s Modified Eagle Medium
DTT	Dithiothreitol
ECM	Extracellular matrix
ELISA	Enzyme-linked immunosorbent assay
ERK	Extracellular signal-regulated kinases
FBS	Fetal bovine serum
FEV_1_	Forced expiratory volume in one second
*FN*	Fibronectin gene
FVC	Forced vital capacity
GM-CSF	Granulocyte-macrophage colony-stimulating factor
HS	Healthy subjects
hTERT	Human telomerase reverse transcriptase
IL	Interleukin
IR	Index of reactivity
ITS	Insulin-Transferrin-Selenium
JNK	c-Jun N-terminal kinases
K2EDTA	Ethylenediaminetetraacetic acid
MAPK	Mitogen-activated protein kinases
mRNA	Messenger ribonucleic acid
MMP	Matrix metalloproteinase
qRT-PCR	Quantitative real-time polymerase chain reaction
PBS	Phosphate buffer saline
PD_20A_	The provocative dose of allergen causing a 20% drop in FEV_1_
PD_20M_	The provocative dose of methacholine causing a 20% drop in FEV_1_
RGDS	Arginyl-glycyl-aspartyl-serine peptide (Arg-Gly-Asp-Ser)
ROS	Reactive oxygen species
RNA	Ribonucleic acid
SD	Standard deviation
SEM	Standard error of the mean
*TGF-β1*	Transforming growth factor β1 gene
TGF-*β*1	Transforming growth factor β1
TSLP	Thymic stromal lymphopoietin
*WNT-5A*	Wingless integrase-1 5A gene
WNT-5A	Wingless integrase-1 5A
